# Novel Epigenetic Target Therapy for Prostate Cancer: A Preclinical Study

**DOI:** 10.1371/journal.pone.0098101

**Published:** 2014-05-22

**Authors:** Ilaria Naldi, Monia Taranta, Lisa Gherardini, Gualtiero Pelosi, Federica Viglione, Settimio Grimaldi, Luca Pani, Caterina Cinti

**Affiliations:** 1 Institute of Clinical Physiology, Consiglio Nazionale delle Ricerche (CNR), Experimental Oncology Unit, Siena, Italy; 2 Institute of Clinical Physiology, Consiglio Nazionale delle Ricerche (CNR), Pisa, Italy; 3 Institute of Translational Pharmacology, Consiglio Nazionale delle Ricerche (CNR), Rome, Italy; 4 Institute of Translational Pharmacology, Consiglio Nazionale delle Ricerche (CNR), Cagliari, Italy; Thomas Jefferson University, United States of America

## Abstract

Epigenetic events are critical contributors to the pathogenesis of cancer, and targeting epigenetic mechanisms represents a novel strategy in anticancer therapy. Classic demethylating agents, such as 5-Aza-2′-deoxycytidine (Decitabine), hold the potential for reprograming somatic cancer cells demonstrating high therapeutic efficacy in haematological malignancies. On the other hand, epigenetic treatment of solid tumours often gives rise to undesired cytotoxic side effects. Appropriate delivery systems able to enrich Decitabine at the site of action and improve its bioavailability would reduce the incidence of toxicity on healthy tissues. In this work we provide preclinical evidences of a safe, versatile and efficient targeted epigenetic therapy to treat hormone sensitive (LNCap) and hormone refractory (DU145) prostate cancers. A novel Decitabine formulation, based on the use of engineered erythrocyte (Erythro-Magneto-Hemagglutinin Virosomes, EMHVs) drug delivery system (DDS) carrying this drug, has been refined. Inside the EMHVs, the drug was shielded from the environment and phosphorylated in its active form. The novel magnetic EMHV DDS, endowed with fusogenic protein, improved the stability of the carried drug and exhibited a high efficiency in confining its delivery at the site of action *in vivo* by applying an external static magnetic field. Here we show that Decitabine loaded into EMHVs induces a significant tumour mass reduction in prostate cancer xenograft models at a concentration, which is seven hundred times lower than the therapeutic dose, suggesting an improved pharmacokinetics/pharmacodynamics of drug. These results are relevant for and discussed in light of developing personalised autologous therapies and innovative clinical approach for the treatment of solid tumours.

## Introduction

Prostate cancer (PC) is a commonly diagnosed malignancy in developed countries and its incidence dramatically increases with age [1], [2]. Despite the proven success of hormonal therapy, surgical castration [3], [4] and radiation therapy [5]–[7], however after being managed a subset of patients manifest disease progression and became resistance to further hormonal manipulation [8]–[10]. Up to 20% of PC patients treated with radical prostatectomy have the probability to progress to invasive cancer with relapsed metastatic conditions within 5 to 10 years [11]. The median survival of patients with metastatic PC is 12–16 months from the time of diagnosis to death [12]. No curative treatments are available at this stage of the disease. In spite of intensive research efforts, the molecular mechanisms by which prostate cancer cells become resistant to hormone therapy remain poorly characterized.

Epigenetic alterations, involving hypermethylation of genes in critical pathways, such as DNA repair, metabolism, and invasion/metastasis, have been found in prostatic cancer providing new information of the pathogenesis of this tumour [13], [14]. Post-translational changes weakening chromatin structure alter gene expression and lead cells to metastatic spreading. However, as epigenetic modifications do not require changing in DNA sequence they are potentially reversible. DNA methyltransferases (DNMTs) involved in the epigenetic silencing of gene expression become therefore a suitable target for epigenetic treatments [15]–[17].

Decitabine (5-Aza-2'-dC) is a classic demethylating agent approved by FDA for the treatment of patients with myelodysplastic syndromes and Acute Lymphoblastic Leukemia (ALM) [18]. However, the beneficial of Decitabine treatment remains uncertain for patients with solid tumours as the lack of chemical stability in aqueous solution and high incidence of neutropenia have been associated with its use.

Recently the anti-proliferative effect of Decitabine has been tested in different tumour histotypes such as testicular [19], lung [20], breast [21], colorectal cancer cells [22], CNS tumours [23] and prostate cancer [24]–[26]. These encouraging results indicated that Decitabine could be effective in inhibiting cancer progression and inducing cell differentiation. Notwithstanding these *in vitro* reports, it was emphasised the need for improving drug stability in solution and delivery efficacy, to minimise toxic side effects and prolong epigenetic outcomes *in vivo*.

The remarkable therapeutic potential of Decitabine is in fact hampered by its systemic instability [27]. Its low therapeutic index and the difficulty to calibrate steady state blood concentration have affected several clinical studies.

In recent years drug delivery systems (DDSs) have been developed to improve the specific and localized delivery of therapeutic agents to target tumour tissue while avoiding severe toxic side effects on healthy organs [28], [29]. Cell-based drug delivery systems such as erythrocytes are particularly attractive tools for delivering several classes of therapeutics. Erythrocytes are safe and biocompatible carriers that can accommodate molecules of different size, nature and stability and they are particularly useful for those agents that show limited tissue penetration or are rapidly inactivated upon i.v. *in vivo* administration [30]–[32]. Moreover, erythrocytes also act as circulating bioreactors converting a pro-drugs into its active forms.

A novel erythrocyte-based drug delivery system, endowed with both super-paramagnetic nanoparticles (NPs) inside the erythrocytes and a fusogenic glycoprotein, the filamentous hemagglutinin (FHA), inserted into the cytoplasmic membranes of erythrocytes (Erythro-Magneto-Hemagglutinin Virosomes, EMHVs), was recently patented (WO2010/070620(A1)).

It has been shown that these EMHV improved kinetics of therapeutic compounds into target cells *in vitro*. Efficient drug intracellular release is achieved into host cells due to presence of fusogenic glycoprotein on EMHV membranes [33], [34].

The magnetic nature of this drug delivery system allows EMHV to be directed toward the desired tissues/organs *in vivo* upon the application of an external magnetic field.

Here we tested a target “epigenetic therapy” based on use of low dose 5-Aza-2'-dC loaded into EMHVs in two prostate cancer models. Human prostate adenocarcinoma LNCap cells, responsive to hormone therapy, and DU145, hormone refractory cells, have been used [35] *in vitro* and *in vivo* in xenograft tumour models. We show that the pharmacological anticancer activity of 5-Aza-2′-dC is highly increased by our EMHVs delivery system both *in vitro* and *in vivo* suggesting its possible application in future clinical trials.

## Materials and Methods

### Reagents

Superparamagnetic nanoparticles (NPs) were purchased from nano-screenMAG, Chemicell, Berlin, Germany. Filamentous Hemagglutinin from *Bordetella pertussis* (FHA), 5-aza-2′-deoxicytitidine (5-Aza-2'-dC), HPLC grade acetonitrile, N′,N′-dimethylhexylamine (DMHA), cytidine-5′-triphosphate disodium salt (CTP) and 2'-deoxyuridine (dU) were purchased from Sigma-Aldrich, Milan, Italy. Methanol and ammonium acetate were purchased from Carlo Erba, Milan, Italy.

### Preparation of 5-Aza-2'-dC-loaded EMHVs (A-EMHVs)

Human erythrocytes were prepared by gradient centrifugation at 400 g for 30 minutes and then washed twice in 1X PBS (1.37 M NaCl, 57 mM KCl, 54 mM Na_2_HPO_4_, 45 mM KH_2_PO_4_ pH 7.4). 2×10^9^ erythrocytes were lysed in 250 µl lysis buffer 1 (10 mM TRIS, 0.1 mM EDTA, 1 mM MgCl_2_ pH 7.2) for 60 minutes at 0°C. The isotonicity was then restored by adding 130 µl of resealing buffer (65 µl of 10X PBS pH 7.4 and 65 µl of 15 mM MgCl_2_ pH 7.4), supplemented with 2 µg of FHA, 0.1 mg of 100 nm super-paramagnetic NPs and 75 µg of 5-Aza-2'-dC.

The suspension was incubated for 45 minutes at 37°C under mild agitation to promote resealing and obtain engineered erythrocytes loaded with 5-Aza-2′-dC (A-EMHVs). A-EMHVs were then collected by centrifugation at 8,000 g for 15 minutes at 4°C. Successively the erythrocyte suspension was washed twice with 1X PBS by centrifugation at 8,000 g for 15 minutes at 4°C, re-suspended in 1X PBS and conserved at 4°C until used.

### Cell culture

LNCap and DU145 prostate cancer cell lines were obtained from American Type Culture Collection (ATCC, Rockville, MD) and maintained in culture medium RPMI 1640 supplemented with 10% Fetal Bovine Serum, 2 mM L-glutamine, in presence of 100 U/ml penicillin-streptomycin, at split ratio of 1∶3 twice a week. These prostate cancer cells were used for both *in vitro* and *in vivo* experiments.

### Confocal Laser Scanning Microscopy (CLSM) analysis

DU145 cells at a density of 1.5×10^5^ were seeded in 6-well microtiter plates. On the bottom of the culture plates glass coverslips were placed on which the cells were let to grow until 60–80% confluence. After 24 hours, the culture medium was replaced with media containing 1.5×10^8^ A-EMHVs. In a sample, fresh medium was replaced with no adding of EMHVs to visualize naïve cell structure (control). After 6, 24 and 48 hours of incubation, coverslips were retrieved and cells were washed in 1X PBS buffer and fixed with 4% paraformaldehyde. Cell nuclei were counter stained with DAPI, washed with 1X PBS and the whole coverslip is mounted on a slide using anti-fade medium. Fluorescence and bright-field images were captured by CSLM, Leica TCS SP5 inverted microscope system, equipped with sources emitting from the UV to the visible. DAPI fluorescence was detected using excitation at 405 nm and recording emission at 454 nm while the red fluorescence of super-paramagnetic NPs was excited at 543 nm and its emission was detected at 613 nm.

### Liquid chromatography–mass spectrometry (HPLC-MS) analysis of 5-Aza-2'-dC loaded into EMHVs

To quantify the total amount of 5-Aza-2'-dC inside the modified erythrocytes, 2×10^9^ freshly prepared A-EMHVs were lysed in 200 µl of lysis buffer 2 (155 mM NH_4_Cl, 10 mM KHCO_3_, 0.1 mM EDTA, pH 7.2) for 10 minutes at room temperature and then centrifuged at 12,000 g for 15 minutes at 4°C. The supernatant was transferred into a Microcon centrifugal filter device with MWCO 30,000 Da (Amicon YM-10, Millipore, Vimodrone MI, Italy) and then centrifuged at 14,000 g for 2 hours at 4°C. Filtered samples were kept at −80°C until analysis. The HPLC system used was a Dionex 3,000 Ultimate series LC (Sunnyvale, CA, USA) connected to a linear ion trap LTQ-Orbitrap mass spectrometer (Thermo Fisher Scientific, USA), equipped with an electrospray ion source. Data were acquired and processed with Excalibur 2.1 software. Compounds were separated on a Mediterranea Sea18 reverse-phase column (150 mm, 2.1 mm I.D. and 5 µm particle size) from Teknokroma (Analytical Technology S.r.l., Brugherio Milan, Italy). The column was set at a flow rate of 0.25 ml/minute, at a temperature of 36°C, and sample volumes of 10 µl were injected. The mobile phase consisted of 5 mM ammonium acetate (solvent A) and acetonitrile (solvent B). The first 13 minutes were an isocratic run with solvent A; between 13 and 20 minutes the percentage of mobile phase B was increased to 35%, maintained for 2 minutes and then the initial the mobile phase was re-established within 2 minutes. The mass spectrometer was operated in positive electrospray mode and the collision energy was 35 eV. The transitions monitored were m/z 229--->113 for 5-Aza-2'-dC; m/z 219--->103 for guanylurea derivatives; m/z 247--->131 for formulated derivatives of 5-Aza-2'-dC. To search for possible phosphorylated forms of 5-Aza-2'-dC, 2×10^9^ freshly prepared A-EMHVs were left at 37°C for 24 hours. Successively, samples were lysed and filtered as above described and 400 ng of CTP were added to the samples as internal standards. The mobile phase consisted of 20 mM DMHA pH 7.0 (solvent A) and methanol-water 80∶20 (solvent B). The first 12 minutes were an isocratic run with 10% solvent B; between 12 and 15 minutes the percentage of mobile phase B was increased to 80%, maintained for 1 minute and then the initial the mobile phase was re-established within 2 minutes. The mass spectrometer was operated in negative electrospray mode and the collision energy was 15 eV. The transitions monitored were m/z 482--->384 for CTP; m/z 467--->369 for 5-Aza-2'-dC triphosphate; m/z 387--->289 for 5-Aza-2'-dC diphosphate; m/z 307--->208 for 5-Aza-2'-dC monophosphate corresponding to the elimination of a neutral molecule of H_3_PO_4_. For the calibration curve, six different CTP standard solutions were used. The peak area ratio of sample/CTP was used for quantization.

### Cytofluorimetric (FACS) analysis

LNCap and DU145 cells at a density of 1.5×10^5^ were seeded in 6-well microtiter plates for cell cycle assays. After 24 hours, the culture medium was replaced with media containing: no drug (CTRL); free 5-Aza-2'-dC at doses 6.8 µg or 120 ng; 1.5×10^8^ A-EMHVs (containing approximately 120 ng 5-Aza-2'-dC).

After 24, 48 and 96 hours of incubation, control and treated cells were harvested and analysed by FACS. Nuclei were stained with 10 µg/ml propidium iodide (PI) in hypotonic solution (1X PBS containing 0.1% sodium citrate and 0.1% Triton X-100) for 30 minutes at 4°C in the dark for assessment of cell cycle phases.

Apoptotic cells were detected by Annexin V test (BioVision). The treated and untreated cells were suspended in 1X binding buffer and incubated at room temperature for 15 minutes with Annexin V-FITC and Propidium Iodide (PI) was added for nuclei staining following manufacturer's instructions.

Flow-cytometry was carried using Becton–Dickinson FACScan CentroII and data were analysed by FlowJo software.

### Animals

For the experiments carried out at the animal facility of Marshall University, congenital athymic BALB/c Nude mice, homozygous for the *nu*/*nu* allele, were bred in house. The colony of 8- to 12-week-old male mice was developed from breeding stock obtained from Charles Rivers Laboratories (Wilmington, MA). These animals were used for LNCap xenograft experiments.

Animals used in experiment carried out at “Toscana Life Sciences” animal facility were athymic Nude-Foxn1*nu* male mice, 6 weeks of age, purchased from Harlan Laboratories (Udine, Italy). These animals were used for DU145 xenografts.

In both cases, animals were housed in micro-isolators in autoclaved cages with polyester fiber filter covers, under germ-free conditions. All food, water, and bedding were sterilized and the animals were maintained in an ambient temperature of 23±2°C in rooms having a 12 hours light/dark cycle.

### X-ray imaging

Athymic BALB/c Nude mice were injected with 1.5×10^8^ EMHVs suspended in 150 µl of 1X PBS.

Following EMHVs tail vein injection, one group of animal were exposed to external magnetic field by placing two earth magnets on the lower lateral abdominal region for 30 minutes (EMHVs-MF), while mice were kept under 2% isofluorane anesthesia. Neodinium magnets (round shaped 5.0 mm) with approximately coercive force of 1,000 KOersted, were used. A control group of mice was tail vein injected with EMHVs as previously described but no external magnetic field was applied (EMHVs-NMF). One hour after the treatment, mice were sacrificed by CO_2_ asphyxiation and the accumulation of ferrous beads was assessed by X-ray imaging. The imaging analysis was performed using a Philips DigitalDiagnost direct digital radiography system with flat detector technology (Philips, Hamburg, Germany) with a dose of 60 kVp at 5 mAs.

### Tumour xenograft procedures

Both athymic BALB/c Nude and Nude-Foxn1*nu* mice were anesthetized by 2.5% isoflurane during manipulation.

The setting of the models: LNCap or DU145 cells were concentrated to 7×10^6^ or 4.5×10^6^ respectively in 1X PBS and injected subcutaneously 1∶1 with MatrigelTM basement membrane matrix (BD Biosciences, Franklin Lakes, NJ) into the left flank of each mouse (total volume 200 µl). Once xenografts started growing, their sizes (mm^3^) were measured twice a week with digital caliper and the volume was calculated by using the standard formula: length x width^2^/2. Tumour xenografts were allowed to grow approximately up to 100 mm^3^ and this volume was selected as the initial stage for beginning the treatment. Mice were randomized (n = 6), anesthetized and prepared for tail vein injection with the selected treatment. Before each injection, tumours were measured and compared to the corresponding initial volumes, in order to normalize the data (X = 100 x volume_1_/volume_0_). Randomisation: Mice were assigned to seven different groups in both experimental settings (LNCap or DU145 xenografts) and they were treated as follow: 1X PBS (CTRL); 85 µg 5-Aza-2'-dC (2.5 mg/kg) (A1); 120 ng 5-Aza-2'-dC (A2); 1.5×10^8^ unloaded EMHVs in absence of static magnetic field (EMHVs-NMF); 1.5×10^8^ unloaded EMHVs and static magnetic field applied on tumour (EMHVs-MF); 1.5×10^8^ EMHVs containing 120 ng 5-Aza-2'-dC (A-EMHVs-NMF); 1.5×10^8^ EMHVs containing 120 ng 5-Aza-2'-dC and static magnetic field applied on tumour (A-EMHVs-MF).

Injection protocol: Mice were biweekly administered intravenously with 150 µl treatment per inoculation, over 3 weeks. After each injection in those groups selected to be treated also with static magnetic field (EMHVs-MF and A-EMHVs-MF), two earth magnets (1,000 KOersted) were applied to the xenograft mass for 30 minutes to ensure intra-tumour accumulation. Mice were then allowed recovering and monitored for signs of distress. At the end of the treatment course or when the tumour volume reached approximately 400 mm^3^, mice were sacrificed by CO_2_ asphyxiation and the prostate tumour mass, liver, kidneys were harvested and stored at -80°C until additional analysis.

### Morphological and immunohistochemical analysis of tumour xenografts

Frozen samples stored at -80°C were equilibrate at −20°C overnight before sectioning by cryostat (Microm HM 500 W) following embedding in OCT.

Consecutive serial cryosections of 5–6 µm thickness were obtained from the middle (largest) portion of each samples, placed on positively charged slides, dried in air for a few minutes and then fixed with cold acetone. Two-four sections were stained with Mayer's hematoxylin for histopathologic examination.

After washing in 1X PBS and incubation in H_2_O_2_ at room temperature to block endogenous peroxidase, the section were incubated with diluted normal blocking serum prepared from the species in which the secondary antibody is made. Slides were incubated in humid chamber overnight at 4°C with primary human reactive antibodies for Ki67 (clone SP6, rabbit monoclonal antibody, Thermoscientific) at a dilution 1∶200 and DNMT3b (clone 52A1018, mouse monoclonal antibody, IMGENEX) at the dilution 1∶150. Following 30 min secondary biotinylated antibody and 30 min Vectastain Elite ABC reagent the slides were then incubation with peroxidase substrate solution (DAB). Slides were counterstained with Mayer's hematoxylin for 1 min, and mounted in aqueous Mount Quick (Bioptica). Microscopic examination was carried out from 2x to 40x magnification under an Olympus BX43 light microscope interfaced to a videocamera for digital acquisition and imaging analysis (DP20 Olympus). The values of DNMT3b and Ki67 positive nuclei were expressed as percentage of positive or negative cells over the counting at least 500 total cells at 20x original magnification.

### Statistical analysis

The cell cycle phases are expressed as mean ± SD of at least n = 3. Three-Ways ANOVA were applied to compare the effect of different treatments (CTRL, 6.8 µg 5-Aza-2'-dC; 120 ng 5-Aza-2'-dC; 1.5×10^8^ A-EMHVs) on cell cycle phases (sub G1, G0-G1, S, G2-M) and One-Way ANOVA was used to compare the effect of A-EMHVs treatment at selected time points (24, 48 and 96 hours).

The apoptotic cells were expressed as mean ± SEM of at least n = 3. Statistical analysis was performed with One-Way ANOVA independently for early and late apoptosis on log transformed data to improve normalization. Pairwise comparisons were tested using Tukey's honestly significant difference criterion.

The *in vivo* results are expressed as mean ± SEM of n = 6. One-Way ANOVA was applied to compare the tumour mass reduction in the evaluation of the xenograft implant after selected treatment using data collected at the last injection. To describe tumour mass reduction rate (50%) in mice after selected treatments, Kaplan-Meier analysis was applied followed by log-rank test.

### Ethics statements

Human red blood cells were obtained from transfusion bags collected from anonymous healthy voluntary donors, which have given their written informed consent carried out in accordance with Italian Government law. It was not necessary the approval from an institutional review board (ethics committee) since neither direct human participation nor involvement of human studies have been foreseen in this work. Samples have been provided by Azienda Ospedaliera Universitaria Senese.

The preclinical study was carried out in strict accordance with the recommendations in the Guide for the Care and Use of Laboratory Animals of the International guidelines on handling of laboratory animals and applying the 3Rs to experiments (in accordance with NIH and European Commission recommendations).

The protocols for Animal Experiments were approved by the Ethics Committee of the Marshall University (Huntington, WV, USA) (Permit Number: # 458/2010) and by the Ethics Committees of the Toscana Life Sciences and the Istituto Superiore di Sanità (ISS) on behalf of Italian Minister of Health (Permit Number: # CNR-270111 exp1/prot1). Animal well-being was monitored accordingly to Langford et al, 2010. [36] "

## Results

### Characterization of novel anticancer formulation in erythrocyte-based drug delivery system

To enhance the performance profile of 5-Aza-2′-dC pro-drug, with respect to chemical stability, pharmacokinetics and pharmacodynamics, engineered magnetic erythrocytes carriers (EMHVs) were used as drug delivery system (DDS). The kinetics of EMHV DDS internalization and its toxicity as well as the efficiency and efficacy of this novel anticancer 5-Aza-2′-dC formulation were tested firstly *in vitro* in hormone sensitive (LNCap) and resistant (DU145) prostate cancer cells.

#### Internalisation of the EMHVs carrier into tumour cell *in vitro*

**Figure 1:**
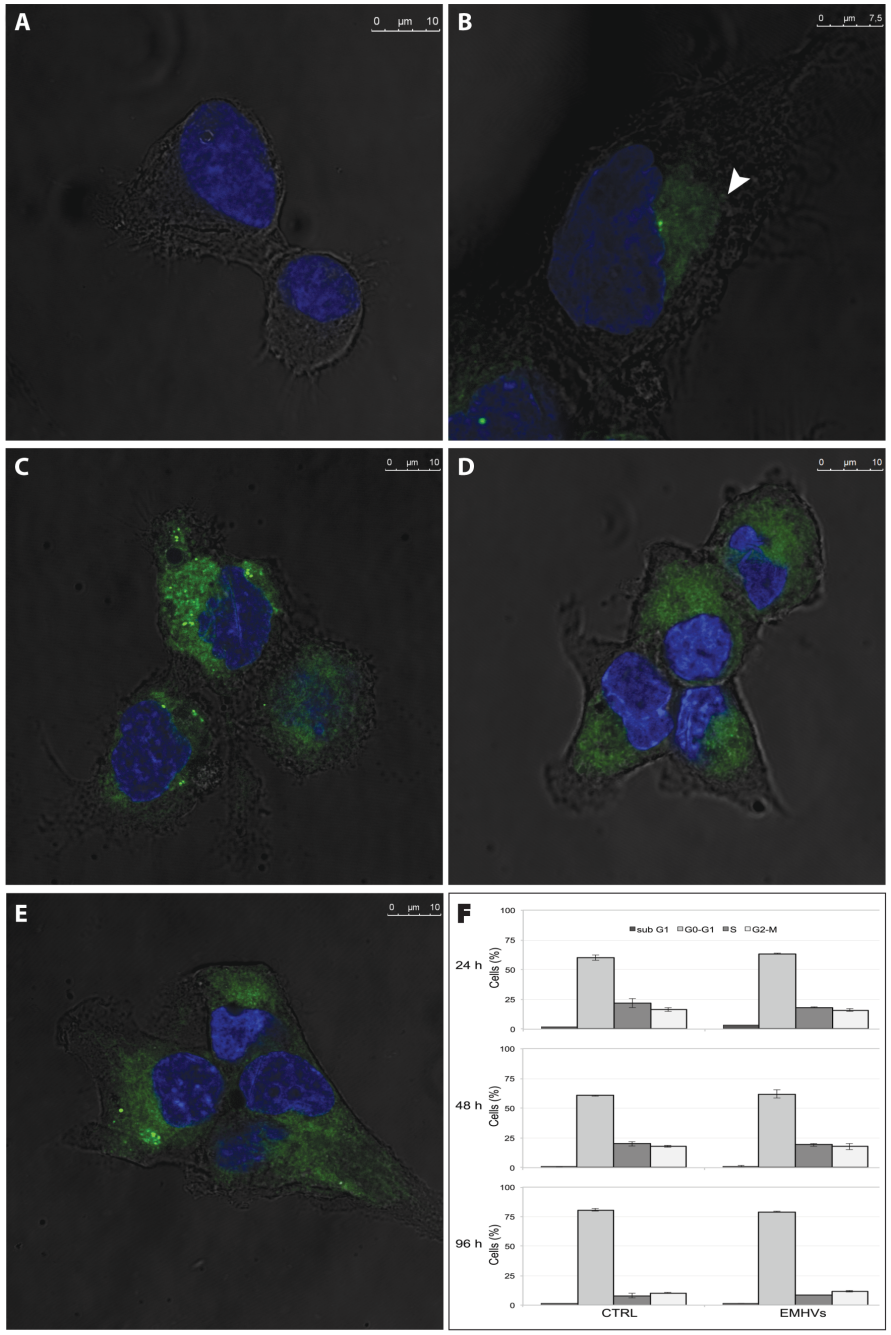
Qualitative and quantitative analysis of the distribution and effect of EMHVs treatment in DU145 prostate cancer cells. Representative CLSM images of EMHVs internalization and released nanoparticle distribution (green fluorescence signal, **B–E**) into the host cell cytoplasm at several time points are depicted. Naïve cells (**A**) are reported as control. In (**B**), after 6 hours of treatment, an intact EMHV is present in the cytoplasm (arrow). At 24 hours (**C**) the particles appeared to have been released from the EMHVs. In the pictures taken at 48 (**D**) and 96 (**E**) hours, nanoparticles appear to have homogeneously distributed throughout the cytoplasm. Lack of toxic effect of erythrocyte drug delivery system treatment was evaluated by FACS analysis (**F**) where cell profile of the treated cells (EMHVs) at selected time points show no significant changes in sub-phase distributions with respect to control (CTRL).

The kinetic of EMHVs internalization into both LNCap and DU145 prostate cancer cells was confirmed by Confocal Laser Scanning Microscopy analysis (CSLM) by monitoring the distribution of released fluorescent NPs (green) into the cytoplasm of target cells (Fig. 1). An example of DU145 naïve cells is shown in Fig. 1A. Internalization is already taking place at 6 hours after treatment, when EMHVs were found inside the cytoplasm of DU145 cells. At this stage EMHVs still retain their intact membrane and their content of NPs (Fig. 1B). Similarly to what found in our previous work [33], after internalization the EMHV membrane fuses with membrane of host cell releasing the fluorescent content in the whole cytoplasmic space (Fig. 1C–E). At the latest time points (48–96 hours) a wide spread fluorescence of NPs is detectable in the cytoplasm although host cells appear morphologically healthy and no cytotoxic effect is visible (Fig. 1D–E).

#### Lack of toxicity of the EMHV carriers on cell models

The toxicity of EMHV DDS against host cells was assessed by analysing cell cycle phases of target cells. When treated with unloaded EMHVs for 48 and 96 hours, no shift in LNCap and DU145 cell phase distribution was detectable and cells retained their normal cell cycle for the duration of the treatment (Fig. 1F). This finding suggests that EMHVs drug delivery system did not exert any toxic effect on cancer cells per se.

#### Chemical stability of 5-Aza-2'-dC into EMHV bioreactor system (A-EMHVs)

5-Aza-2'-dC is a pro-drug and it requires to be activated by phosphorylation to a nucleoside triphosphate before exerting inhibition on DNA methylation [37].

**Figure 2:**
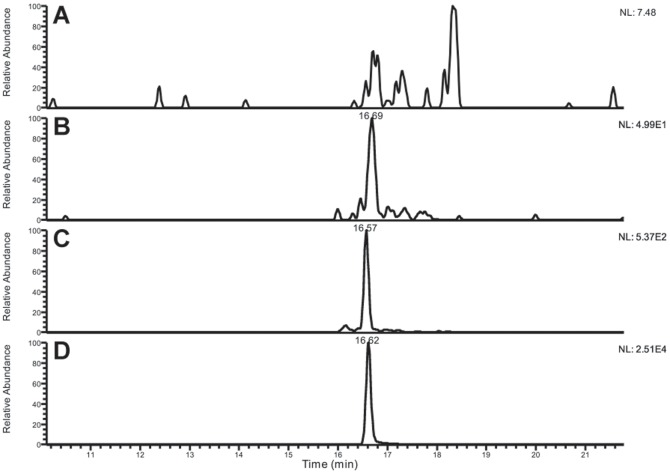
HPLC-MS chromatogram of the phosphorylated forms of 5-Aza-2′-dC. (**A**) chromatograms for 5-Aza-2′-dC mono-phosphate; (**B**) di-phosphate (RT: 16.6); (**C**) tri-phosphate (RT: 16.5) and (**D**) internal standard CTP (RT: 16.5).

Recently it has been shown that erythrocytes act as bioreactors due to their enzymatic systems [34]. This makes them suitable for encapsulation of pro-drugs that will subsequently be transformed into the active drug [38]. Loading 5-Aza-2'-dC pro-drug into EMHV bioreactor system was achieved according to a standardized protocol (see materials and methods). HPLC-MS was used to quantify the total amount of drug inside the DDS (A-EMHVs) and to detect the presence of its active phosphorylated forms. The chromatogram of samples incubated for 24 hours at 37°C highlighted a mixture of different phosphorylated forms of 5-Aza2′-dC (Fig. 2). Di- and tri-phosphate forms of phosphorylated 5-Aza2′-dC elute at RT 16.6 and RT 16.5 respectively (Fig. 2B and 2C). Figure 2D shows the peak of cytidine thriphosphate (CTP; RT: 16.5), an internal standard that we added to our samples as control. It appears that the tri-phosphate form of 5-Aza2′-dC overbears the other phosphorylated forms proving the high efficiency of EMHVs as bioreactors. We found that 1.5×10^8^ A-EMHVs accommodates about 120 ng of total drug and that the active phosphorylated forms of 5-Aza-2'-dC represents the 50% of total loaded drug into the A-EMHVs.

#### The anti proliferative activity of A-EMHVs *in vitro*

The anti-proliferative effect of A-EMHV in inducing cell cycle arrest and apoptosis was tested in both prostate cancer models: hormone sensitive LNCap cells and androgen independent prostatic cancer DU145 cell line where traditional therapeutic approaches are hampered by the lack of responsiveness to hormone therapy and PSA antigen expression on the cell membrane.

The effect of 1.5×10^8^ A-EMHVs treatment, containing approximately 120 ng internal 5-Aza2′-dC, has been compared with that of free 5-Aza-2'-dC used at the dose of 2.5 µM (total of 6.8 µg) scaled down from the therapeutic dose used in clinics. We also compared the effect of the same amount of 5-Aza-2′-dC contained inside the A-EMHVs (120 ng) used as free drug solution.

**Figure 3:**
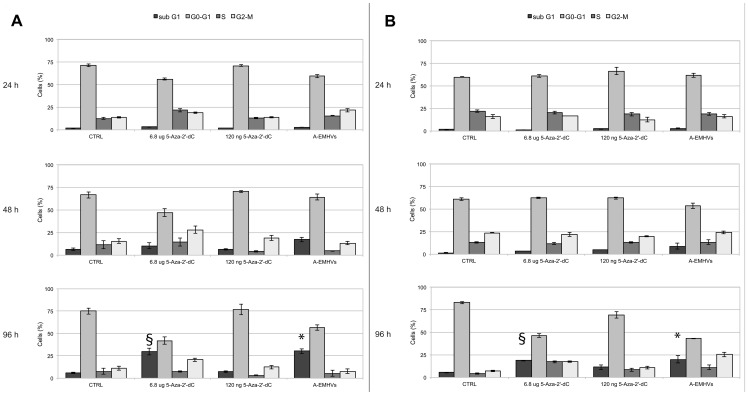
The effect of A-EMHVs on the cell cycle arrest of LNCap, hormone sensitive and DU145 hormone refractory cells. 1.5×10^8^ A-EMHVs treatment, containing 120 ng internal 5-Aza-2'-dC, was compared with 6.8 µg free 5-Aza-2'-dC and 120 ng free 5-Aza-2'-dC treatments at 24 (top) 48 (middle) 96 (bottom) hours. Untreated cells were used as control (CTRL). In both LNCap (**A**) and DU145 (**B**) cell lines, A-EMHVs treatment induced a significant enrichment in sub G1 cell distribution (black bars) already detectable at 48 hours after treatment (**A** and **B** middle panel, respectively) that last up to 96 hours (*ANOVA p<0.05). Similar shift toward sub G1 distribution was also obtained using 6.8 µg of free 5-Aza-2'-dC (§ANOVA p<0.05) but only detected at 96 hours. No shift in cell cycle distribution was detected for free 120 ng 5-Aza-2'-dC, not differing from controls.

On hormone responsive LNCap cells (Fig. 3A), the A-EMHVs treatment induced a significant enrichment in sub G1 suggesting a possible activation of apoptotic response (Fig. 3A). This shift was already detectable at 48 hours after treatment (Fig. 3A middle panel) and reached statistic significance at 96 hours (ANOVA p<0.05). A similar shift toward sub G1 distribution was obtained also using 6.8 µg of free 5-Aza-2'-dC (ANOVA p<0.05), however this dose is more than 50 times higher than the 5-Aza-2'-dC loaded into the A-EMHVs. Moreover, the effect of 6.8 µg free 5-Aza-2'-dC treatment seems to have a later onset comparing to A-EMHVs. This is probably due to the fact that 5-Aza-2'-dC, encapsulated into the EMHVs is readily turned into phosphorylated forms improving 5-Aza-2'-dC pharmacokinetics/pharmacodynamics. Notably low doses of free 5-Aza-2'-dC (120 ng) did not exert sub G1 shift effect and the cell cycle distribution profile did not differ from controls up to 96 hours.

A similar trend towards sub G1 phase was described in hormone resistant DU145 cell line (Fig. 3B) after either treatment with A-EMHVs or 6.8 µg free 5-Aza-2'-dC at 96 hours (Fig. 3B lower panel, ANOVA p<0.05) while no change in cell cycle was detected after lower free 5-Aza-2'-dC treatment at any time points. Because a hypermethylation of some tumour suppressor genes was found in DU145, this suggests that a reprogramming of these drug resistant cells towards susceptibility of cells to apoptosis was achieved following epigenetic therapy.

**Figure 4:**
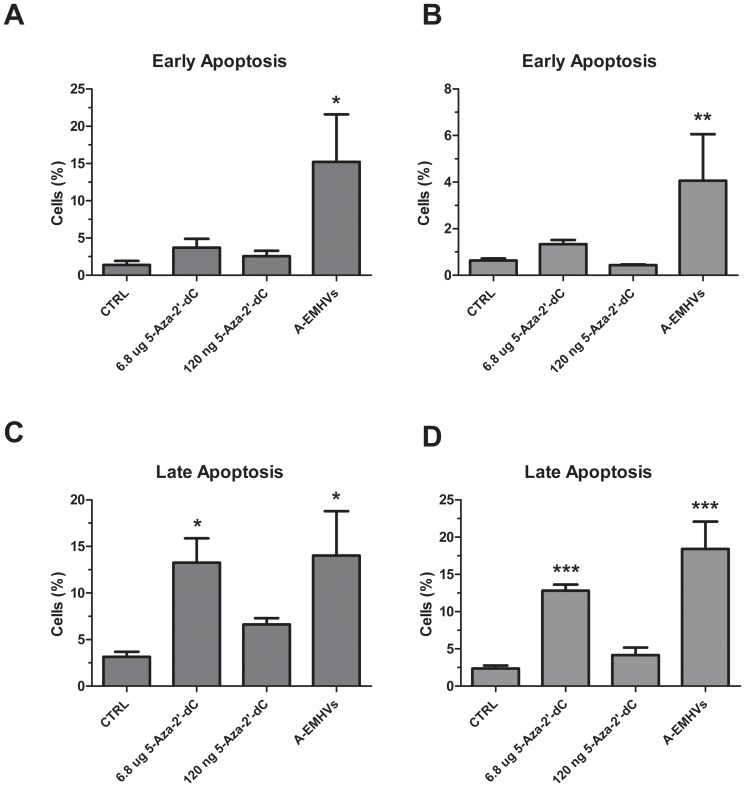
The pro-apoptotic effect of A-EMHVs on LNCap, hormone sensitive and DU145, hormone refractory cells. 1.5×10^8^ A-EMHVs treatment, containing 120 ng internal 5-Aza-2'-dC, was compared with 6.8 µg free 5-Aza-2'-dC and 120 ng free 5-Aza-2'-dC treatments at 96 hours. Untreated cells were used as control (CTRL). In both LNCap (**A**) and DU145 (**B**) cell lines, A-EMHVs treatment induced a significant enrichment in early apoptosis (*ANOVA p<0.05 and **ANOVA p<0.01, respectively). Using 6.8 µg or 120 ng of free 5-Aza-2'-dC, no effect in early apoptosis was detected in any cell lines at 96 hours. Significant increase in late apoptosis response was observed in LNCap (**C**) and DU145 (**D**) after treatment with A-EMHVs as well as with 6.8 µg of free 5-Aza-2'-dC (*ANOVA p<0.05 and ***ANOVA p<0.001, respectively), whereas no change in late apoptosis was detected after lower free 5-Aza-2'-dC treatment at 96 hours in any of the cell lines.

To assesse if the treatments can induce the activation of pro-apoptotic response, Annexin V test has been performed. The analysis indicates that A-EMHVs exert a pro-apoptotic effect in both prostate cancer cell lines (Fig. 4). In particular the A-EMHVs treatment induced enrichment in early and late apoptosis at 96 hours after treatment (Fig. 4A and 4C) on LNCap cells, reaching statistic significance in both cases (ANOVA p<0.05). A similar result, but only for late apoptosis, was obtained using 6.8 µg of free 5-Aza-2'-dC (ANOVA p<0.05), whereas the level of early apoptosis, induced by treatment, does not statistically differ from control. A similar trend for both early and late apoptosis was described in hormone resistant DU145 cells (Fig. 4B and 4D), after treatment with A-EMHVs at 96 hours (ANOVA p<0.01 and p<0.001, respectively). Using 6.8 µg of free 5-Aza-2'-dC, a significant increase in late apoptosis was obtained, while no effect was detected for early apoptosis. No change in apoptosis was detected after lower free 5-Aza-2'-dC treatment at 96 hours in either of the cell lines.

### Characterization of novel anticancer formulation *in vivo*

To set-up an efficient and effective target epigenetic therapy for solid tumours and overcome the obstacles represented by the use of demethylating agents in clinical practice [39], [40], we investigated the therapeutic potential of 5-Aza-2′-dC (Decitabine) in a novel anticancer formulation *in vivo*.

#### *In vivo* selective concentration of EMHV by magnetic field

To verify that systemic intravenous (i.v) administration of A-EMHVs could benefit the localised treatment of solid tumour, we first assessed if EMHV DDS could be efficiently driven to reach selected body compartments. Naïve animals were i.v. injected with 1.5×10^8^ EMHVs. Immediately after injection a group of animals was exposed to an external static magnetic field applied for 30 minutes to their lower lateral abdominal region (EMHVs-MF). Control animal only received the injection and no magnetic field was applied (EMHVs-NMF).

**Figure 5:**
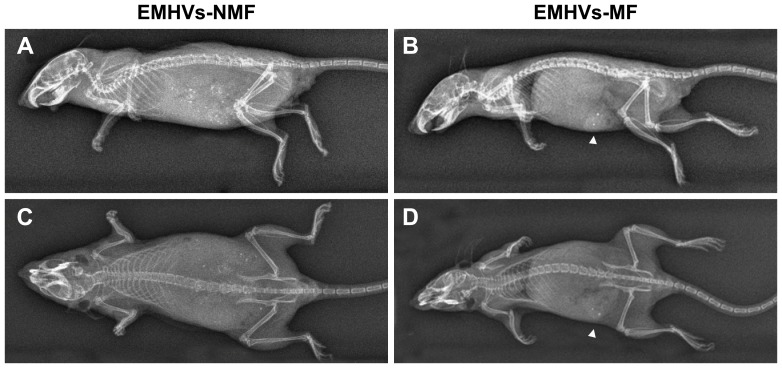
Localized targeted in vivo distribution of EMHVs after systemic tail vein administration in mice. Representative X-ray images of mice injected with EMHVs and treated with null (**A** and **C**) or 1,000 KOersted external static magnetic field (**B** and **D**) applied for 30 min in correspondence of the abdomen, are depicted. After 1 hour from the injection, a rather sparse distribution of nanoparticles (white irregular shadow/dots) was found inside metabolic organs and tissues exposed to null magnetic field (EMHVs-NMF, **A** sagittal and **C** frontal planes, respectively). After exposure to the magnetic field (EMHVs-MF, **B** sagittal and **D** frontal planes, respectively), nanoparticles concentrated in the target tissue (abdomen) resulting in a sharp intense signal (arrow).

X-ray imaging, acquired 1 hour after the injection, shows specific localization of the EMHVs in a mouse treated with EMHVs in presence of magnetic field (Fig. 5B sagittal plane and Fig. 5D frontal plane representative pictures). This animal displays few well-defined localized bright spots in the abdomen, in correspondence to the site of magnet application (1,000 KOersted). No specific accumulation was evident in the control, treated in absence of the magnetic field (EMHVs-NMF Fig. 5A and 5C, sagittal and frontal planes, respectively), where a rather sparse distribution of nanoparticles was found in the abdomen area, apparently proximal to the liver and kidneys organs.

#### Effect of A-EMHVs on human hormone responsive LNCap prostate cancer xenograft models

**Figure 6:**
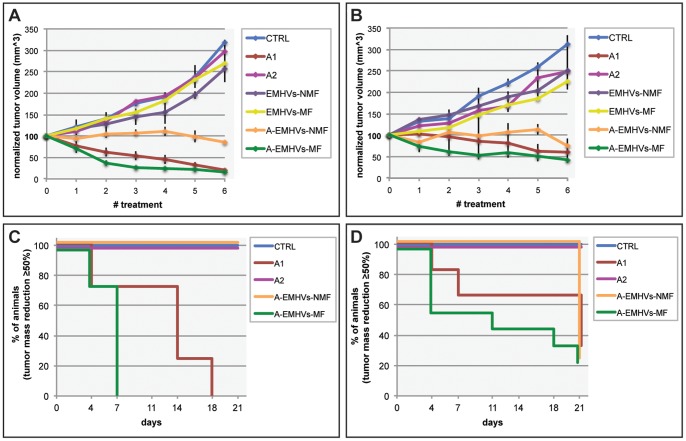
The anticancer effect of A-EMHVs treatment *in vivo*. Mass tumour growth curves for all experimental group are depicted in panel **A** and **B**. The treatments are Control (CTRL, cyan); therapeutic dose (A1, red); low dose equivalent to that inside the erythrocytes (A2, purple); EMHVs and no magnetic field applied (EMHVs-NMF, blue); EMHVs undergoing magnetic field (EMHVs-MF, yellow); loaded EMHVs and no magnetic field applied (A-EMHVs-NMF, orange); loaded EMHVs undergoing magnetic field application (A-EMHVs-MF, green). In (**A**) LNCap xenograft, the most significant mass reduction was measured both in A-EMHVs-MF and A1, while other treatments exerted intermediate effect with respect to controls (One-Way ANOVA p<0.05 at time of the sixth injection). In (**B**) DU145 xenograft, mass showed a similar trend with A-EMHVs-MF and A1 treatments being the most effective (One-Way ANOVA p<0.05). In **C** and **D**, Kaplan-Meier estimator curve was used to determine the percentage of treated mice showing a reduction of tumour mass volume (≥50%) during treatment. In (**C**) the trend of reduction is depicted for LNCap xenograft model: 100% of animals show ≥50% tumour volume reduction after A-EMHVs-MF (day 7^th^) and A1 (day18^th^) treatments. In DU145 xenograft model (**D**), A-EMHVs-MF (green) is the most effective anti-tumour treatment (e.g. CTRL or A2 vs A-EMHVs-MF, log rank = 4.52 at day 21^st^).

The therapeutic potential of the novel 5-Aza-2'-dC anticancer formulation (A-EMHVs) was investigated *in vivo* on animals bearing flank cancer xenografts. In LNCap xenografts (Fig. 6A), a constant increased tumour mass volume was measured in control animals (CTRL) as well as in the groups treated with unloaded 1.5×10^8^ EMHVs either in absence (EMHVs-NMF) or presence (EMHVs-MF) of static magnetic field. In CTRL, volume reached 318.10±5.90 mm^3^, while in EMHVs-NMF and EMHVs-MF reached 257.80±31.78 and 268.84±7.72 mm^3^ respectively, confirming that unloaded EMHVs delivery system does not induce any cytotoxic effect on target tissues per se (see also Fig. 1F, *in vitro* data). Interestingly, also the lower dose of free 5-Aza-2'-dC, (120 ng, A2), similar to those entrapped into A-EHMVs, did not alter tumour mass growth that increased up 296.19±21.55 mm^3^, similar to controls.

On the contrary, a constant reduction of LNCap tumour mass growth was observed in both A1 group treated with 85 µg free 5-Aza-2'-dC (2.5 mg/kg) and the group treated with A-EMHVs in presence of magnetic field (A-EHMVs-MF). More specifically, all A-EMHVs-MF animals showed a reduction in tumour volume as early as the second/third injection (A-EMHVs-MF 35.91±6.29 mm^3^ and 27.18±3.97 mm^3^) and this trend was maintained up to the latest 6^th^ injection (A-EMHVs-MF 15.96±2.77 mm^3^). Similarly, a significant volume reduction was measured in A1 group after fourth/fifth injection (A1 45.24±10.94 mm^3^ and 21.35±6.50 mm^3^) and this trend was maintained up to the latest 6^th^ injection (A1, 20.63±4.59 mm^3^). Therefore, although A-EMHVs-MF contains 700 times lower dose (120 ng) than A1 therapeutic dose (85 µg/i.v.), it results significantly more effective than the therapeutic dose (Two-Ways ANOVA RM p<0.01).

In absence of static magnetic field (NMF), A-EMHVs treatment exerts a strong cytostatic action, inhibiting the tumour growth already after the first up to the latest injection (A-EMHVs-NMF 84.87±2.09 mm^3^), although its effect is significantly lower in respect to A-EMHVs-MF and A1 treatment (Two-Ways ANOVA RM p<0.001).

#### The effect of A-EMHVs on non-responsive prostate cancer DU145 xenograft model

Similarly to what described *in vitro*, DU145 xenograft model is responsive to anti-cancer treatments (Fig. 6B). As expected DU145 tumour mass grew steadily during the entire duration of the experiment in CTRL, EMHVs-NMF and EMHVs-MF groups (values at sixth injection: CTRL 312.54±28.23 mm^3^; EMHVs-NMF 250.86±19.25 mm^3^; EMHVs-MF 224.50±10.85 mm^3^). Moreover, also the low amount of free 5-Aza-2'-dC was not sufficient to stop tumour growth (value at sixth injection A2 group 248.94±70.25 mm^3^). Nonetheless, tumour volume reduction of *circa* 50% was visible after the third injection of A-EMHVs in presence of magnetic field (A-EMHVs-MF 51.65±8.65 mm^3^) up to the last measurement at time of the sixth injection (A-EMHVs-MF 40.43±5.29 mm^3^). Interestingly, this reduction was not visible in A1 group. In the case of DU145 hormone resistant cells, treatment with therapeutic dose (85 µg/injection) of free 5-Aza-2′-dC (value at sixth injection A1 group 75.82±21.9 mm^3^) was significantly less effective than carrier delivered therapy, although the amount of injected free 5-Aza-2-dC was 700 times higher with respect to those contained in A-EMHVs as verified by Two-Ways ANOVA RM (A-EMHV-MF versus and A1, p<0.05).

A-EMHVs in absence of magnetic field (value at time of sixth injection A-EMHVs-NMF 127.88±17.59 mm^3^) only exerted a slight cytostatic activity on tumour mass and was significantly less effective than A-EMHV-MF (Two-Ways ANOVA RM: A-EMHV-NMF vs A-EMHV-MF p<0.001) and A1 treatment (Two-Ways ANOVA RM: A-EMHV-NMF vs A1 p<0.05).

#### Estimation of mass reduction rate *in vivo*

The introduction of the magnetic field therefore re-enforces the anticancer effect of A-EMHVs (A-EMHVs-MF vs A-EMHVs-NMF). This is depicted in the Kaplan-Meier estimator curve for both prostate cancer xenograft models (Fig. 6C and 6D). We chose ≥50% tumour volume reduction as the significant event to estimate and analyse the percentage of animals that demonstrated this reduction in time course.

In A-EMHVs-NMF as well as A2 and CTRL groups, the treatments are not able to exert a beneficial reduction ≥50% of tumour volume, neither in LNCap nor DU145 xenograft models.

A strong anti-cancer effect of the treatments was detected in both A-EMHV-MF and A1 groups of LNCap xenograft as 100% animals showed reduction of their mass volume more that 50% at the 7^th^ day and 18^th^ day, respectively (log-rank test >3.84 A-EMHV-MF or A1 vs CTRL). Similar although milder anticancer therapeutic effect was measured in DU145 xenografts with 78% of animals showing reduction of tumour volume of ≥50% in A-EMHVs-MF group (log-rank test >3.84 A-EMHVs-MF vs CTRL) and the 50% of A1 animals at day 21^st^. See also Figures S1 and S2 showing representative images of mice bearing tumours and intact severed specimens.

Therefore, comparing the new anticancer formulation with the free administration of therapeutic dose either in hormone sensitive and resistant forms of prostate cancer, the effect of this novel Decitabine formulation on tumour volume reduction seems to exert a stronger and faster effect with respect to drug free (A1) therapy although the amount of 5-Aza-2′-dC contained into the formulation in each injected dose is much lower that the free administered drug.

#### Histological and immunohistochemical evidence of epigenetic A-EMHV-MF treatment

A-EMHVs-MF induced the most significant morphological changes in the tumour mass severed from xenograft implants when compared with CTRL or the equal dose (120 ng) of free 5-Aza-2'-dC treatment (A2). A first qualitative analysis of tumour mass sections indicates that the A-EMHVs-MF treatment induced a visible reduction of the slice volume occupied by tumour cells associated with an extensive replacement of death cells by fibrous tissue around the residual tumour mass. Some fibrocytes and fibroblasts were visible within the capsular tissue surrounding the tumour mass as well as replacing the necrotic tumour cells.

**Figure 7:**
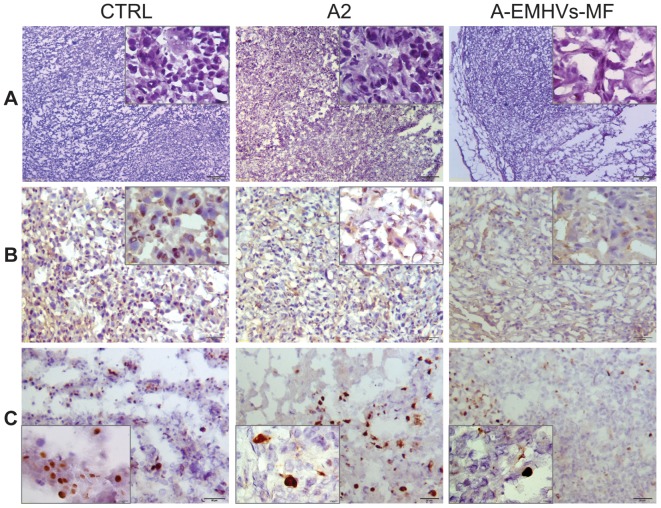
Histological and immunohistochemical features of explanted xenografts. Representative sections of controls (CTRL), 120 ng of free 5-Aza-2'-dC (A2) and A-EMHVs-MF treated animals. Top panels (**A**) show H&E stain at low power (2x original magnification) and high power (40x original magnification, insets) micrographs. In **B**, immunohistological reactivity, positive for nuclear DNMT3b methyltrasferase (DNA methyltransferase 3b) as a marker of tumour cells de novo methylation is shown (positive nuclei: dark brown, negative nuclei: blue. Original magnification 10x and insets at 40x). In the bottom panels (**C**) proliferating tumour cells are visualized using Ki67 marker (positive nuclei: dark brown, negative nuclei: blue. Original magnification 10x and insets at 40x).

A thorough investigation using standard H&E stain confirmed that A-EMHVs-MF was the most effective treatment in promoting tumour cells death as highlighted by loss of nucleic staining (right inserts) and fibrous repair (Fig. 7 panel A). On the other hand, no microscopically detectable changes in sparse fibrocytes and fibroblasts were found across CTRL and A-EMHVs-MF samples stained with H&E, suggesting that the A-EMHVs-MF treatment did not induced any significant damage or toxic site effect on normal cells.

As to confirm the epigenetic nature of the therapy, a higher immunoreaction positivity for DNMT3b (DNA methyltransferase 3b) was found in tumour cells nuclei of control animals, (15.7% positivity of a cumulative number of 595 cells, Table 1) (Fig. 7 panel B, CTRL), and in animals treated with 120 ng free 5-Aza-2'-dC (14.7%, of 651 cells; Fig. 7 panel B, A2) as compared to animals treated with A-EMHVs in presence of MF (10.4% of 619 cells; Fig. 7 panel B, A-EMHVs-MF). These results suggest that loading 5-Aza-2'-dC into the erythrocytes (120 ng per dose) leads to a more efficient inhibition of DNMT3b and in turn to increased rate of tumour cell death. These data correlate with the observation that nuclear immunoreactivity of the Ki67 cell proliferating marker (Fig. 7 panel C) markedly decreases after A-EMHVs-MF treatment (6.5% positive nuclei of a total nuclear count of 572 cells) as compared to CTRL and A2 (20.3% and 16.8% positive nuclei, respectively), suggesting that A-EMHVs-MF hampers cell replicating activity within tumour mass.

**Table 1:** Evaluation of cell proliferation after treatments.

	CTRL		A2		A-EMHVs-MF	
	absolute value	%	absolute value	%	absolute value	%
**DNMT3 +**	93	15,7%	76	14,7%	64	10,4%
**DNMT3 −**	502	84,3%	575	88,3%	555	89,6%
total cell count	**595**		**651**		**619**	
**Ki67 +**	124	20,3%	95	16,8%	37	6,5%
**Ki67 −**	488	79,7%	469	83,2%	535	93,5%
total cell count	**612**		**564**		**572**	

Immuno-histochemical positivity for DNMT3b and Ki67. The results are expressed as cumulative number of and the percentage of reactive cells. Controls samples showed the highest number of reactive cells in comparison to either A2 or A-EMHVs-MF treatments.

All together the *in vivo* data indicate that the administration of 5-Aza-2′-dC-loaded EMHVs in mice could be considered as a locally effective epigenetic therapy. In fact it induced a significant improvement in the pharmacokinetics of drug given that low dosage of the demethylating agent was sufficient to reduce expression of DNMT and to reprogram cell fate.

## Discussion

The potential of novel target epigenetic therapy, based on the use of engineered magnetic erythrocyte (EMHVs) drug delivery system, has been demonstrated in this work both *in vitro* and *in vivo* in hormone sensitive and resistant prostate cancer models opening new therapeutic prospective for its future clinical application against other solid tumours.

Using EMHVs, we were able to maximise the anti-cancer effectiveness of 5-Aza-2'-dC demethylating agent and to improve the bioavailability as well as pharmacokinetic and pharmacodynamics of drug. 5-Aza-2'-dC loaded into the carriers exhibited a significant anti-proliferative effect comparable only to the use of much higher doses of free Decitabine. This effect is likely to be related to the phosphorylation of Decitabine, critical step for anticancer activity occurred inside the EMHVs that acted as bioreactors as demonstrated by HPLC analysis. The shield provided by EMHVs to the phosphorylated active form of drug protects it from host enzyme degradation and promotes the pro-drug to active drug transition. This would improve Decitabine transient therapeutic effect and reduce inter-patient variability to therapy response [41]. This is in good agreement with recent data on Decitabine-loaded nanogels synthesized to deliver low amount of drug to chemo-resistant breast cancer cells *in vitro*. In this work the prolonged anti-proliferative effect of Decitabine was ascribed to the sustained delivery rate achieved with the use of nanogels that sustained DNMT1 depletion [21].

*In vivo* however, Decitabine epigenetic prolonged effect is strictly dose schedule dependent and drug concentration and bioavailability influence administration regime [27], [39]. Here we were able to further improve Decitabine pharmacokinetics *in vivo*, by confining and concentrating the circulating drug-loaded EMHVs at the site of action through the application of external static magnetic field on animal xenograft tumours.

As shown in *in vitro* experiments, the fate of drug-loaded EMHVs, reaching their target, is to fuse with and release their content directly inside the host cells.

Kaplan-Meier estimator analysis demonstrated in fact a highly significant reduction in growth of tumour mass in animals treated with loaded EMHVs and magnetic field application, when compared with free Decitabine treatment. This result derived from two synergistic effects: the initial burst of delivery driven by the magnetic field application and the subsequent cytostatic effect that drug-loaded EMHVs provided as they remained in the blood pool acting as a circulating reservoir of active drug that continued to exert its anticancer activity after magnetic field removal.

The safety of the EMHVs delivery system was ascertained neither *in vitro* nor *in vivo* as undesired toxic effect was detected. In previous work we demonstrated that EMHVs are indistinguishable from host erythrocytes therefore physiological RES processing is awaiting EMHVs that might not be held by the applied magnetic force. Toxicity tests i*n vivo* demonstrated the safety of EMHVs carrier in immune competent mice [34]. Thus the use of loaded EMHVs would provide a low dose, safe targeted therapy effect probably reducing possible toxic side-effects, such as granulocytopenia often observed in patients treated with Decitabine.

Moreover, the magnetic nanoparticles within engineered erythrocytes are approved by FDA for use with Magnetic Resonance Imaging (MRI) as a contrast agent to provide an improved image of body organs and tissues in diagnostic routine. It has been shown that these nanoparticles are eliminated predominantly via the kidneys and their use appear in general to be safe and well tolerated [42]. We demonstrated that EMHVs carrying magnetic nanoparticles could be concentrated into selected superficial or internal body districts by means of application of a biocompatible external magnetic field thus reducing the risk factors that could emerge by intravenous administration of free magnetic nanoparticles. This no invasive application uses similar magnetic force field employed in MRI practice, suggesting the possibility of developing loaded EMHVs formulation as a specific locally driven theranostic agent, thus opening new therapeutic prospective for the use of 5-Aza-2′-dC-EMHVs formulation in other solid tumours.

Because aberrant DNA methylation associated with inappropriate gene silencing is a common feature of solid tumours, DNA methylation inhibitors might contribute an alternative therapy especially for those tumours no responsive to conventional chemotherapy. Recent works identified aberrant methylation of some genes in LNCap and DU145 cells and highlight the potential therapeutic value of demethylating agents in prostate cancer that poor respond to conventional therapy [43], [44].

Notably, we were able to tackle proliferation in prostate hormone resistant cancer cells suggesting that the epigenetic mechanisms are pivotal in development of resistant phenotype. Future work should involve screening of reprogrammed methylation and reactivated gene expression in this unresponsive model to correlate with anti-proliferative profile, which in turn would result in halting of cancer metastatic fate. Although the exact anticancer mechanism of 5-Aza-2′-dC is controversial, however our results suggest its possible role in the reduction of DNMT expression. The majority of the reports used somatic cells to assess anticancer activity of Decitabine at high dosage. There is evidence that incorporation of 5-Aza-2′-dC in DNA lead to the sequestration of DNMT enzymes and in turn to hypomethylation of specific genes suppressing DNA repair activity [45]. Recently, a specific sensitivity to low 5-Aza-2′-dC dose (10 nM) by human embryonic carcinoma cells (EC) was described in testicular germ cell tumours. More specifically, hypersensitivity was correlated with high expression of DNMT3b proper of embryonic and cancer stem cells. In these cells, the early effect on DNMT3 depletion and gene methylation was evident within 72 hours of treatment [19]. Similarly we found that the anti-proliferative effect due to loaded EMHVs administration (120 ng 5-Aza-2′-dC) in both LNCap and DU145 was significantly higher at 24 and lasted up to 96 hours in culture. A-EMHVs treatment resulted in a fast and strong shift of cell cycle phase distribution towards sub G1, inducing the cell cycle arrest. In fact apoptotic response was stronger after A-EMHVs treatment, comparing free 5-Aza-2′-dC conventional treatment, likely due to the fact that EMHVs directly release and enriched the therapeutic phosphorylated active drug inside the cells.

Moreover, 5-Aza-2'-dC was also found to regulate gene re-expression in a DNA methylation-independent manner through the breaking up complex protein interactions by inhibition and removal of DNMTs from the nucleus. It is likely that this pronounced A-EMHVs-MF effect on prostate hormone responsive and non-responsive tumour cells could be mediated by the intrinsic direct inhibitory activity exerted by 5-Aza-2'-dC on the DNA methyltransferases [37], [46], [47]. Indeed immunohistochemical findings in specimens from *in vivo* xenografts suggested a specific involvement of DNMT3b inhibition exerted by the low dose 5-Aza-2′-dC-loaded EMHVs (120 ng) in presence of magnetic field causing apoptotic response on target cancer cells. A reduce expression of DNMT3 was in fact found in tumour mass sections after treatment with A-EMHVs-MF, while the same 5-Aza-2′-dC concentration administered free was not able to exert any changes in immunohistochemical staining or cytotoxic effect.

Coincidently the expression of Ki67 pro-proliferative factor is highly down regulated in tumour cells of animal group treated with A-EMHVs-MF, suggesting that reprogramming of the tumour suppressor gene methylation might take place and re-establishing chemotherapy sensitivity would prompt those cancer cells to respond to a subsequent conventional chemotherapy. Interestingly, no morphological damages were visible in fibrocytes and fibroblasts surrounding and infiltrating tumour mass suggesting the A-EMHVs-MF treatment did not exerted any local toxicity to normal cells.

EMHVs are versatile carriers that have been used to target the delivery of different types of molecule and for the treatment of diseases other that cancer. Recently, their use for a gene therapy in a vascular restenosis models has been reported [48]. Similarly, in this work erythrocyte-based delivery of bio-drugs resulted more efficient than the delivery by other conventional vehicles. Up to now the need for *ex-vivo* manipulations with RBCs, a relatively limited shelf life, concerns related to the safety of donors matching and blood-born infections have limited their use as DDS carriers. The development of a personalized medicine for cancer or other diseases based on the use of autologous loaded EMHVs is getting nonetheless more and more feasible, as progress in modulating the RBC membrane to produce transferable stealth donor RBCs have been made. Indeed, the use of autologous blood (re-infusion) would minimize the safety concerns. Loaded EMHVs may be easily produced on the daily bases by self-donation of patients that could lately receive the therapy with minimal discomfort and total safety. This would encourage the “bench to bedside” translation of innovative clinical approaches.

## Conclusions

The scientific rationale for this investigation is the urgent need to find new safe strategies for tackling common forms of cancer, such as prostate cancer that often develops into metastatic and untreatable forms. Increasing evidence suggests that epigenetic events are causally implicated in prostate cancer development as epigenetic silencing of androgen receptor expression has been observed in 8% of primary prostate cancers. In the present work we exploited the therapeutic potential of classic demethylating agent Decitabine to induce tumour cell reprogramming by loading it into an erythrocyte-based delivery system to improve pharmacokinetics and pharmacodynamics. This approach represents a novel formulation of anticancer treatment that can be easily localised at site of action by a non-invasive safe magnetic system. Interestingly this novel Decitabine formulation might find new application in unresponsive forms of carcinoma, such as hormone refractory prostate cancer. As several challenges exist to successfully translate the outcomes from animal research to humans in a clinical setting we foresee future investigation to confirm EMHVs effectiveness in orthotopic animal model of cancer before beginning studies in patients.

## Supporting Information

Figure S1 **Representative images of mice bearing tumours and intact severed specimens of LNCap xenograft groups after the sixth injection.** **CTRL** animal treated with 1X PBS; **A1** animal treated with 85 µg 5-Aza-2'-dC (2.5 mg/kg); **A2** animal treated with 120 ng 5-Aza-2'-dC; **EMHVs-NMF** animal treated with 1.5×10^8^ unloaded EMHVs drug delivery system in absence of static magnetic field; **A- EMHVs-NMF** animal treated with 1.5×10^8^ EMHVs containing 120 ng 5-Aza-2'-dC in absence of static magnetic field; **A- EMHVs-MF** animal treated with 1.5×10^8^ EMHVs containing 120 ng 5-Aza-2'-dC and static magnetic field applied on tumour.
(TIFF)

Figure S2 **Representative images of mice bearing tumours and intact severed specimens of DU145 xenograft groups after the sixth injection.** **CTRL** animal treated with 1X PBS; **A1** animal treated with 85 µg 5-Aza-2'-dC (2.5 mg/kg); **A2** animal treated with 120 ng 5-Aza-2'-dC; **EMHVs-NMF** animal treated with 1.5×10^8^ unloaded EMHVs drug delivery system in absence of static magnetic field; **A- EMHVs-NMF** animal treated with 1.5×10^8^ EMHVs containing 120 ng 5-Aza-2'-dC in absence of static magnetic field; **A- EMHVs-MF** animal treated with 1.5×10^8^ EMHVs containing 120 ng 5-Aza-2'-dC and static magnetic field applied on tumour.
(TIF)
