# Supplementary figures and images for: Novel Epigenetic Target Therapy for Prostate Cancer: A Preclinical Study
Source: PLoS One. 2014 May 22;9(5):e98101. doi: 10.1371/journal.pone.0098101 (PMC4031137; doi:10.1371/journal.pone.0098101)

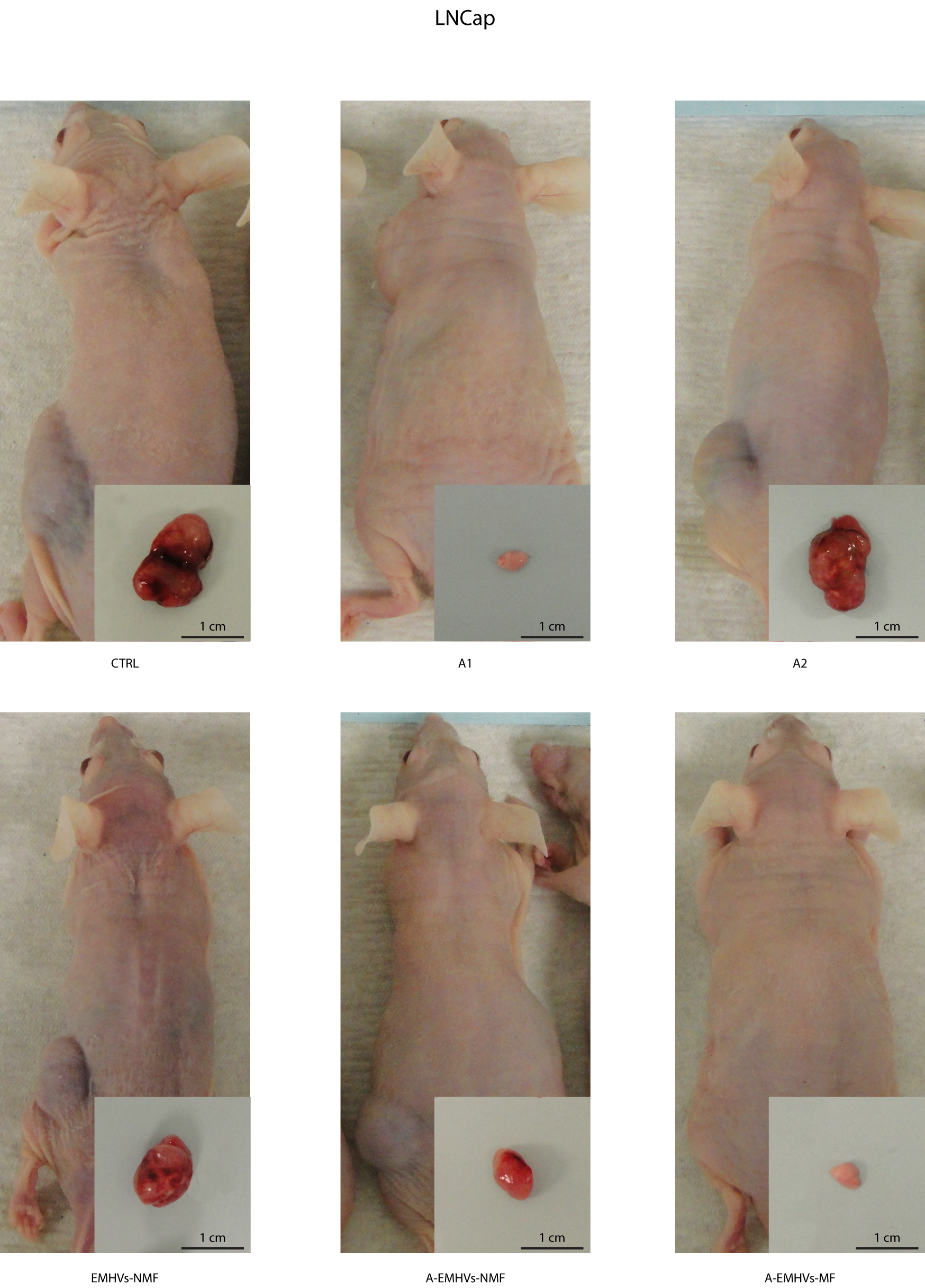

Supplement: Figure S1 — Representative images of mice bearing tumours and intact severed specimens of LNCap xenograft groups after the sixth injection. CTRL animal treated with 1X PBS; A1 animal treated with 85 µg 5-Aza-2'-dC (2.5 mg/kg); A2 animal treated with 120 ng 5-Aza-2'-dC; EMHVs-NMF animal treated with 1.5×10^8^ unloaded EMHVs drug delivery system in absence of static magnetic field; A- EMHVs-NMF animal treated with 1.5×10^8^ EMHVs containing 120 ng 5-Aza-2'-dC in absence of static magnetic field; A- EMHVs-MF animal treated with 1.5×10^8^ EMHVs containing 120 ng 5-Aza-2'-dC and static magnetic field applied on tumour. (TIFF) [file pone.0098101.s001.tif]

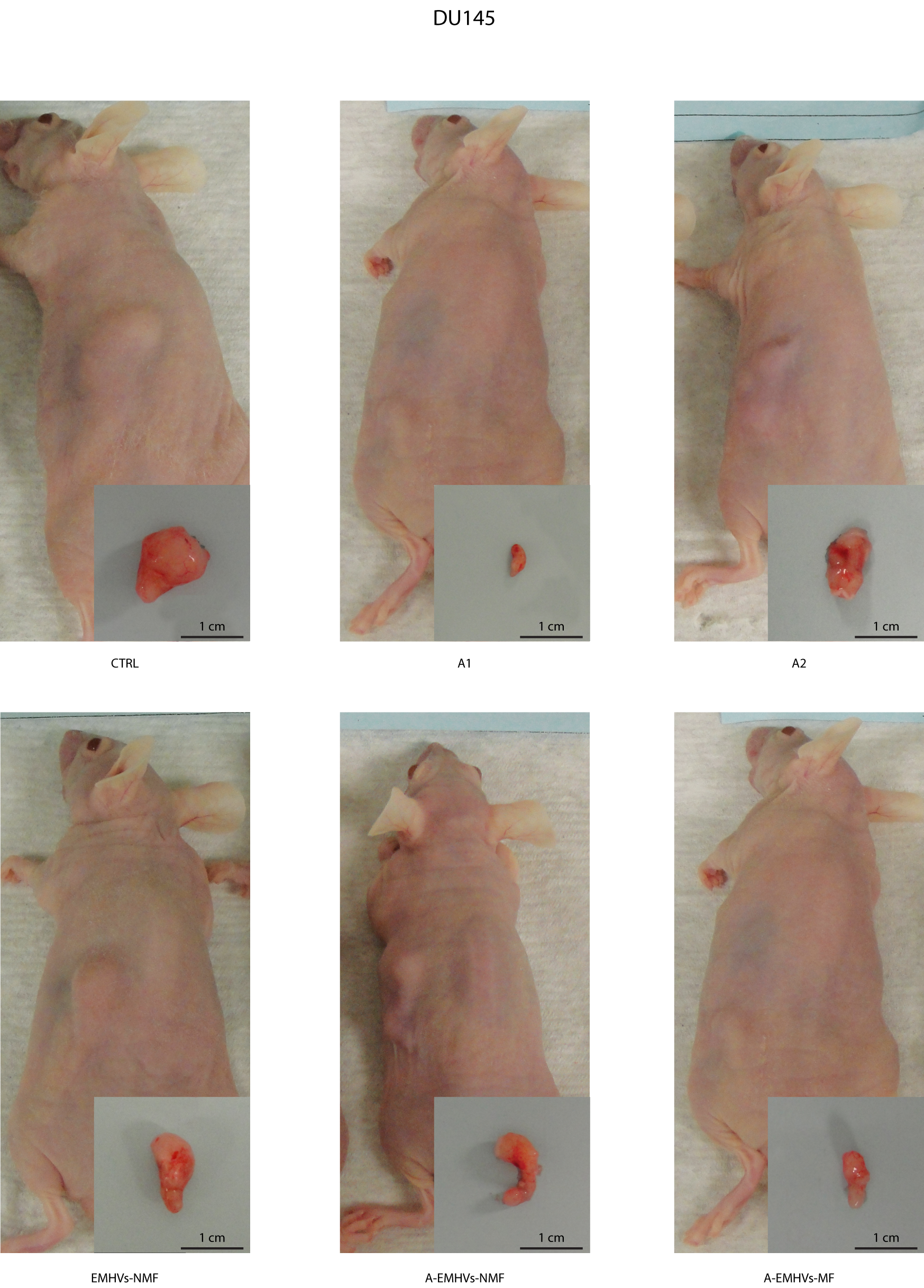

Supplement: Figure S2 — Representative images of mice bearing tumours and intact severed specimens of DU145 xenograft groups after the sixth injection. CTRL animal treated with 1X PBS; A1 animal treated with 85 µg 5-Aza-2'-dC (2.5 mg/kg); A2 animal treated with 120 ng 5-Aza-2'-dC; EMHVs-NMF animal treated with 1.5×10^8^ unloaded EMHVs drug delivery system in absence of static magnetic field; A- EMHVs-NMF animal treated with 1.5×10^8^ EMHVs containing 120 ng 5-Aza-2'-dC in absence of static magnetic field; A- EMHVs-MF animal treated with 1.5×10^8^ EMHVs containing 120 ng 5-Aza-2'-dC and static magnetic field applied on tumour. (TIF) [file pone.0098101.s002.tif]
